# Dosimetric Comparison of 3D Conformal Radiotherapy (3D-CRT), Intensity-Modulated Radiotherapy (IMRT), and Volumetric-Modulated Arc Therapy (VMAT) in Cardiac-Sparing Whole Lung Irradiation

**DOI:** 10.7759/cureus.51047

**Published:** 2023-12-24

**Authors:** Ryohei Yamauchi, Shinobu Akiyama, Norifumi Mizuno, Takako Kobayashi, Tomoko Itazawa, Tomoyuki Masuda, Miki Hirano, Fumihiro Tomita, Yosuke Hosoya, Jiro Kawamori

**Affiliations:** 1 Department of Radiation Oncology, St. Luke’s International Hospital, Tokyo, JPN; 2 Department of Pediatrics, St. Luke’s International Hospital, Tokyo, JPN

**Keywords:** intensity-modulated radiation therapy (imrt), dosimetric comparison study, volumetric-modulated arc therapy, lung metastasis, respiratory motion, whole lung irradiation (wli), ewing sarcoma (es)

## Abstract

Introduction

Whole lung irradiation (WLI) is used for the treatment of lung metastasis in Wilms tumor and Ewing sarcoma; however, cardiac complications are one of the concerns. We report the dosimetric advantages of WLI using volumetric-modulated arc therapy (VMAT) and present a dosimetric comparison of VMAT with anteroposterior-posteroanterior (AP-PA) and static-field intensity-modulated radiation therapy (IMRT). Additionally, we evaluated the dosimetric impact of respiratory motion and intra-fractional motion during VMAT treatment.

Methods

Seven patients were recruited in this study. AP-PA, IMRT, one-isocenter (1-IC) VMAT, and 2-IC VMAT were planned on the maximum inspiration and expiration CT, respectively. The prescribed dose was 15 Gy in 10 fractions. To determine the effects of respiratory motion, the CT series was replaced and the dose was evaluated while maintaining the beam information. To determine the effect of patient motion, perturbed dose calculations were performed using a two-IC VMAT. The perturbation doses were calculated by shifting only the IC of the one side beam by 3 mm or 5 mm in the right-to-left (RL) direction.

Results

The mean heart dose was 1467.0 cGy, 790.0 cGy, 764.2 cGy, and 738.4 cGy for AP-PA, IMRT, 1-IC VMAT, and 2-IC VMAT, respectively. When the expiration CT plan was recalculated with inspiration CT, D_max_ increased approximately by 8%. In the 2-IC VMAT plan, the D_50%_, D_98%_, and D_2%_ dose differences were within ±2%, even with a 5 mm IC shift.

Conclusion

We confirmed a significant dosimetric advantage of VMAT over other techniques. 2-IC VMAT should be considered an effective treatment option during irradiation for large target volumes.

## Introduction

Whole lung irradiation (WLI) is widely used as a treatment option for lung metastasis of Wilms tumor, Ewing sarcoma, and rhabdomyosarcoma in children and adolescents [1-5]. Most studies retrospectively focused on the outcomes and toxicity of WLI, predominantly including children and adolescents; however, some studies report on the feasibility of WLI in adult patients with Ewing sarcoma [6,7]. A traditional delivery technique of WLI uses the anteroposterior-posteroanterior (AP-PA) field; thus, high-dose volumes to the heart cannot be avoided. Cardiac complications, such as congestive heart failure (CHF), myocardial infarction, pericardial disease, and valvular heart disease, are important late effects observed in childhood cancer survivors after WLI [4,8].

Kalapurakal et al. proposed the use of a novel cardiac-sparing intensity-modulated radiation therapy (IMRT) technique to improve dose coverage and spare cardiac irradiation [9,10]. Subsequently, Papachristofilou et al. and Suzuki et al. reported their experiences of WLI using volumetric-modulated arc therapy (VMAT) instead of static-field IMRT, and Suzuki et al. reported that VMAT has superior dose distribution over the traditional AP-PA technique [11,12]. However, as these articles are case reports, they were conducted only on a single patient and have not been validated on multiple patients. Additionally, there are merely any published reports comparing dose distribution between VMAT, AP-PA fields, and static-field IMRT. Dosimetric parameters, such as the target coverage, dose conformity, and homogeneity, are generally equivalent between VMAT and static-field IMRT [13]. However, VMAT potentially offers additional advantages, such as lower treatment delivery time, compared with static-field IMRT, which may increase the use of VMAT for WLI.

Adolescent and adult patients have larger target lungs, which may create mechanical constraints when planning IMRT and VMAT treatments. For example, multileaf collimators (MLC) on Varian linear accelerators cannot travel 14.5 cm past the central axis. Although some treatment planning systems automatically split large IMRT fields into several subfields, the limitations of splitting the IMRT field include an increase in the total monitor units (MU), number of treatment fields, and treatment time. One solution is to limit the size of the irradiation field; however, this may lead to degradation of dose distribution. Therefore, a two-isocenter (2-IC) VMAT with ICs in both lungs was proposed as a method to solve these issues, and its feasibility was examined. Another issue with WLI is that no studies have reported dose effects from respiratory motion due to the small number of patients and the difficulty of conducting a detailed study.

The aim of this study is to clarify the superiority of VMAT, including the proposed 2-IC VMAT, by comparing the dose distributions with AP-PA and static-field IMRT, and to evaluate the effect of different respiratory phases on dose distribution in VMAT plans.

## Materials and methods

CT simulation and normal tissue and target volume definitions

A 14-year-old male patient with metastatic Ewing sarcoma was referred to our department for WLI. The study protocol was approved by our institutional review board (22-R074). The CT simulation and treatment were performed in a supine position with both arms raised above the head. The first CT scan was performed using a Siemens SOMATOM Confidence Pro scanner (Siemens Healthineers, Germany) without gating devices. During the simulation, 2 mm thick CT slices were obtained from the mandible to the pelvic brim. The second CT scan was a four-dimensional-gated CT scan (4DCT) using the Varian real-time position management system (Varian Medical Systems, CA, USA).

We contoured the following organs at risk (OAR): spinal cord, liver, and heart. The lung internal target volume (ITV) was the maximum lung expansion volume defined as the minimum intensity projection bilateral lung volume on all phases of 4DCT. The lung planning target volume (PTV) was obtained by a 0.7 cm expansion of this ITV for the inferior direction and 0.5 cm expansion for the other direction.

As it is a rare treatment with only a small number of cases, a feasibility study was conducted to assess the dose distribution of different delivery techniques in seven patients, including six additional patients. These six patients were selected from those who had chest CT (both free-breathing and 4DCT) and there was no additional exposure for this plan. However, it should be noted that they were not pediatric cancer patients due to the restrictions mentioned above.

Treatment planning

All plans were generated on maximum inspiration CT (i.e., 0% phase CT) and maximum expiration CT (i.e., 50% phase CT) using a radiation treatment system (RayStation, version 9.0; RaySearch Laboratories, Stockholm, Sweden). Here, the target volumes (PTV and ITV) were the same regardless of the respiratory phase being calculated. Dose calculation was performed using a collapsed-cone convolution algorithm with heterogeneity correction and a constant 2 mm calculation grid size. The prescribed dose was 15 Gy in 10 fractions. A 6 MV or 10 MV x-ray linear accelerator was employed (Clinac-iX; Varian Medical Systems).

**Figure 1:**
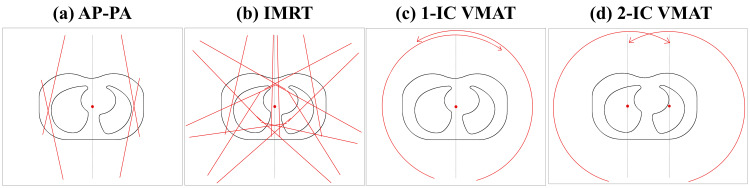
Four plans with different field designs for (a) AP-PA, (b) IMRT, (c) 1-IC VMAT, and (d) 2-IC VMAT Red points indicate ICs, and red lines or arrows indicate irradiation fields or arc settings. AP-PA, anteroposterior-posteroanterior technique; IMRT, intensity-modulated radiation therapy; VMAT, volumetric-modulated arc therapy; IC, isocenter

Four plans with different field designs were created for optimization as shown in Figure 1. First, the standard AP-PA field consists of two equally weighted fields. Second, similar to Kalapurakal’s study, static-field IMRT was used. IMRT plans were performed with seven beams using a dynamic technique with 50 segments per beam, allowing the use of split fields [10]. Third, similar to Bosarge’s study, a partial five-arc VMAT was used. For the right lung treatment, the angle of the two arcs was set to 30° from 181° clockwise and counterclockwise, and collimator angles were set to 15° and 345° [14]. Similarly, for the left lung treatment, the angle of the three arcs was set to 330° from 179° clockwise and counterclockwise, and collimator angles were set to 15°, 345°, and 85°, respectively. Fourth, similar to the third design, the two ICs were used and these ICs moved approximately 5 cm toward the center of each lung. The gantry and collimator angles were similar to the third design. A total of 56 plans (seven patients × four plan designs × two phases) were generated.

For IMRT and VMAT plans, the dose received by at least 50% of the PTV volume was set to 15 Gy. Plan optimization was performed using target and OAR dose constraints based on Kalapurakal’s study. Namely, dose-volume constraints for the heart were as follows: 20%, 40%, 60%, 80%, and 100% dose of the heart were 11.8 Gy, 11 Gy, 10 Gy, 8 Gy, and 4.5 Gy, respectively [10]. The goal of the target was that ≥95% of the PTV should receive ≥95% of the prescribed dose and that ≤1% of the PTV should receive >110% of the prescribed dose. For OAR doses, the maximum doses should be <107% of the prescribed dose.

Dosimetric parameters for the target and OARs were calculated by recording the percentage volume of the target or organs receiving a dose greater than X Gy (V_X Gy_), the volume receiving a dose greater than X% of the prescribed dose (V_X%_), and the dose to percentage volumes (D_X%_). We calculated the homogeneity index (HI) using the ICRU83 equation [15]. Additionally, the target coverage, maximum dose, and uniformity were assessed. Moreover, for OARs such as heart and liver, the mean or volume dose was assessed. Evaluation of the middle-lower dose volumes was performed by computing the volume of the body receiving 80%, 50%, and 30% of the prescribed dose.

Evaluation of dosimetric impact by intra-fractional setup uncertainties

In a multi-IC VMAT, dose uniformity, coverage, and OAR dose inside the junction can be issues because of intra-fractional setup uncertainties. In order to determine the dosimetric impact of intra-fractional motion during treatment, the perturbed dose calculation tool that is inbuilt within RayStation was used on 2-IC VMAT plans. We generated a perturbed dose distribution by shifting the IC of the left lung beams in the right-to-left (RL) direction using this tool. An IC shift of 3 mm and 5 mm was applied. Subsequently, we compared the dosimetric parameters in D_50%_, D_98%_, and D_2%_ for ITV between the original and perturbed dose plans.

Evaluation of dosimetric impact by respiratory motion

The dose impact due to the difference between the respiratory phase used in the treatment planning series and the respiratory phase during treatment was evaluated. While maintaining the beam information, the dose calculation CT series was replaced, and the dosimetry indices were evaluated. In other words, a plan generated with 0% phase CT was recalculated with 50% phase CT and vice versa. We compared the dosimetric parameters in D_50%_, D_98%_, D_2%_, D_max_, and HI for PTV between the original and recalculated dose plans.

Statistical analysis

We employed EZR version 1.37, a graphical user interface for R (R Foundation for Statistical Computing, Vienna, Austria) for statistical analysis. A Friedman test with Bonferroni’s correction was conducted and p-value <0.05 was considered statistically significant.

## Results

Comparison of dosimetric parameters among different field designs

The volumes of the heart, ITV, and PTV for the treated 14-year-old male patient were 458.4 cm^3^, 3294.7 cm^3^, and 4359.8 cm^3^, respectively. The mean (range) volume of the heart, ITV, and PTV for all patients were 671.0 (458.4 - 925.4) cm^3^, 3625.7 (2191.8 - 5670.9) cm^3^, and 4786.6 (3024.5 - 7169.8) cm^3^, respectively. The mean and standard deviation (SD) of total MU was 179.4 ± 5.3, 2248.4 ± 314.6, 1105.4 ± 90.3, and 1006.3 ± 63.6 in the AP-PA, IMRT, 1-IC VMAT, and 2-IC VMAT, respectively.

**Figure 2:**
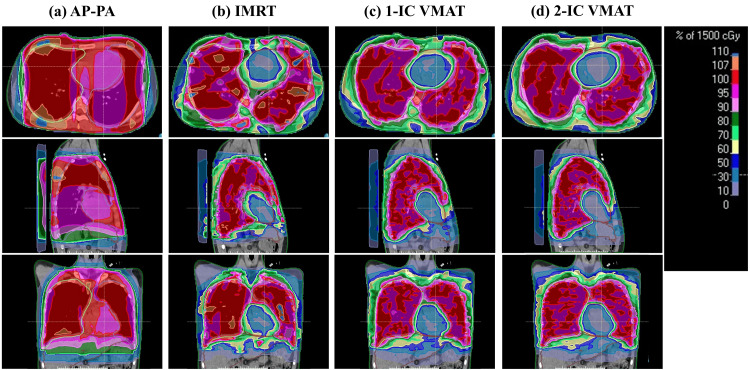
Comparison of dose distribution for (a) AP-PA, (b) IMRT, (c) 1-IC VMAT, and (d) 2-IC VMAT The top, middle, and bottom panel shows the axial, sagittal, and coronal views. The color of the dose distribution is represented as a percentage of the prescribed dose. AP-PA, anteroposterior-posteroanterior technique; IMRT, intensity-modulated radiation therapy; VMAT, volumetric-modulated arc therapy; IC, isocenter

**Figure 3:**
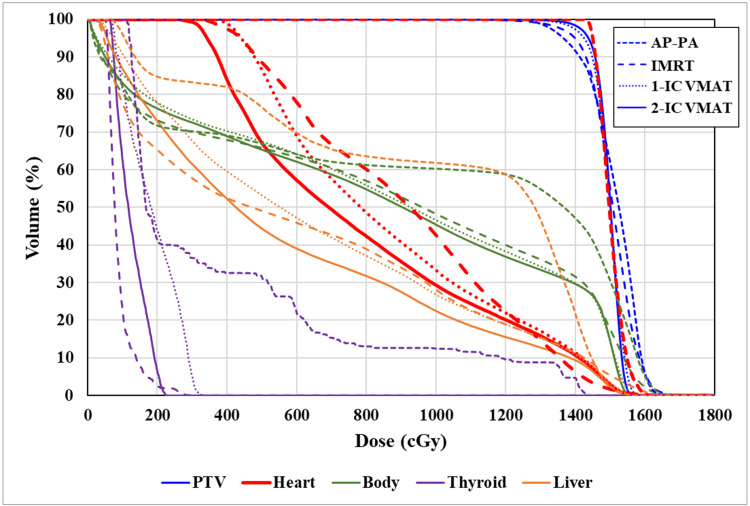
Dose-volume histograms of a patient for four different techniques AP-PA, anteroposterior-posteroanterior technique; IMRT, intensity-modulated radiation therapy; VMAT, volumetric-modulated arc therapy; PTV, planning target volume; IC, isocenter

Figure 2 and Figure 3 show examples of dose distributions due to the difference in delivery techniques and the dose-volume histograms. Table 1 shows dose-volume data of the target and OARs for all plans. The dosimetric evaluation showed that the mean heart dose was 1467.0 cGy, 790.0 cGy, 764.2 cGy, and 738.4 cGy in the AP-PA, IMRT, 1-IC VMAT, and 2-IC VMAT, respectively. The plan quality used by HI was 0.197, 0.136, 0.117, and 0.094 in the AP-PA, IMRT, 1-IC VMAT, and 2-IC VMAT, respectively. Both VMAT plans had significantly better dose distributions than AP-PA and IMRT plans in several dose endpoints, including PTV coverage, the maximum dose, and the heart dose. In addition, we confirmed the dosimetric advantages of 2-IC VMAT over 1-IC VMAT, including superior heart sparing and superior dose homogeneity in the PTV. The percentages of the body receiving 80% of the prescribed dose were also more favorable with 2-IC VMAT when compared with other techniques. On the other hand, there was no significant difference in the percentages of the body receiving 30% of the prescribed dose between different delivery techniques.

**Table 1:** Mean and SD of dose-volume data of the target and OARs for all plans Notes: A Friedman test with Bonferroni's correction was performed, and statistically significant pairs (p-value <0.05) were indicated by symbols as follows: a = AP-PA vs. IMRT; b = AP-PA vs. 1-IC VMAT; c = AP-PA vs. 2-IC VMAT; d = IMRT vs. 1-IC VMAT; e = IMRT vs. 2-IC VMAT; f = 1-IC VMAT vs. 2-IC VMAT. VX Gy, the percentage volume of the target or organs receiving a dose greater than X Gy; VX%, the volume receiving a dose greater than X% of the prescribed dose; DX%, the dose to percentage volumes; Dmean, the mean dose AP-PA, anteroposterior-posteroanterior technique; IMRT, intensity-modulated radiation therapy; VMAT, volumetric-modulated arc therapy; IC, isocenter; HI, homogeneity index; SD, standard deviation

Target/organs	Dosimetric parameters	AP-PA	IMRT	1-IC VMAT	2-IC VMAT	P-value
PTV	D_50%_ (cGy)	1511.4 ± 36.3	1500.6 ± 2.2	1501 ± 1.2	1501.1 ± 1.8	
	V_95%_ (%)	81.6 ± 10.0	95.2 ± 2.0	95.1 ± 1.5	96.9 ± 0.8	a,b,c,e,f
	V_107%_ (%)	5.8 ± 14.3	1.1 ± 1.4	0.0 ± 0.0	0.0 ± 0.0	b,c,d,e
	V_110%_ (%)	1.2 ± 4.0	0.1 ± 0.2	0.0 ± 0.0	0.0 ± 0.0	b,c,d,e
	D_98%_ (cGy)	1311.7 ± 58.2	1386.2 ± 27.7	1387 ± 20.1	1409.8 ± 11.7	a,b,c,e,f
	D_2%_ (cGy)	1608.3 ± 26.5	1590.1 ± 16.5	1562.8 ± 4.6	1551.2 ± 6.3	b,c,d,e,f
	HI	0.197 ± 0.034	0.136 ± 0.026	0.117 ± 0.016	0.094 ± 0.010	a,b,c,e,f
Heart	V_4.5 Gy_ (%)	99.8 ± 0.7	76.5 ± 8.3	75.1 ± 10.6	69.0 ± 8.2	a,b,c,f
	V_8 Gy_ (%)	99.5 ± 1.6	44.7 ± 8.5	37.6 ± 7.6	35.6 ± 6.3	a,b,c
	V_10 Gy_ (%)	99.4 ± 1.8	30.8 ± 7.2	26.2 ± 6.5	25.3 ± 5.8	a,b,c,d,e
	V_11 Gy_ (%)	99.4 ± 1.9	24.1 ± 5.7	22.0 ± 5.7	21.5 ± 5.2	a,b,c,d,e
	V_11.8 Gy_ (%)	99.4 ± 2.0	19.7 ± 4.8	19.2 ± 5.1	18.8 ± 4.8	a,b,c,d,e
	D_mean_ (cGy)	1467.0 ± 54.1	790.0 ± 72.3	764.2 ± 75.6	738.4 ± 64.5	a,b,c,e,f
	D_2%_ (cGy)	1577.7 ± 40.2	1492.6 ± 34.5	1517 ± 30.5	1514.2 ± 29.9	a,b,c,d
Liver	D_mean_ (cGy)	1191.5 ± 148.1	832.2 ± 256.8	878.5 ± 268.8	838.6 ± 263	a,b,c,f
Body	V_12 Gy_ (cm^3^)	66.5 ± 7.5	43.0 ± 9.9	42.6 ± 10	41.3 ± 10	a,b,c,e,f
	V_7.5 Gy_ (cm^3^)	70.2 ± 7.7	67.1 ± 9.2	66.2 ± 9.2	65.6 ± 8.7	a,b,c,e
	V_4.5 Gy_ (cm^3^)	76.7 ± 7.2	79.0 ± 7.6	78.4 ± 7.9	77.9 ± 7.7	

**Figure 4:**
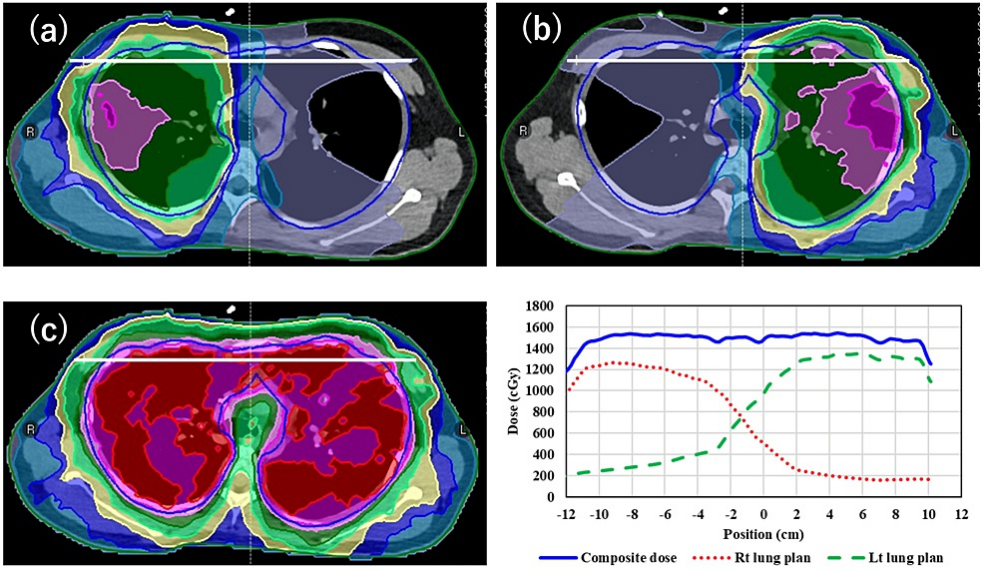
Example of dose distribution for each plan (a, b) and their composite plan (c), and the right bottom shows the line dose of lateral profiles

Figure 4 presents the dose distribution and line dose of the lateral profile for each plan and their composite plan in 2-IC VMAT. Profiles were taken along the center of the PTV from the RL direction through the junction area where the two arcs meet and overlap each other.

Evaluation of dosimetric impact by intra-fractional setup uncertainties

Table 2 shows the mean and SD of the relative difference between the original and perturbed dose plans. The D_50%_ of ITV had a relative dose difference within ±1%, even with a displacement of ±5 mm. When the left and right ICs were closer to each other, the D_2%_ value increased by a maximum of approximately 2%. Conversely, when they were farther apart, the D_98%_ value decreased by a maximum of approximately 2%.

**Table 2:** Mean and SD of the relative difference between the original and perturbed dose 2-IC VMAT plans A positive IC shift value represents the direction of movement toward the opposite IC. SD, standard deviation; D_X%_, the dose to percentage volumes, IC, isocenter; VMAT, volumetric-modulated arc therapy

Dosimetric parameters	IC shift value			
	5 mm	3 mm	-3 mm	-5 mm
D_50%_	0.6 ± 0.2%	0.4 ± 0.2%	-0.2 ± 0.1%	-0.8 ± 0.1%
D_98%_	0 ± 0.2%	0.2 ± 0.1%	-1.1 ± 0.4%	-2.2 ± 0.6%
D_2%_	1.8 ± 0.4%	0.8 ± 0.2%	-0.2 ± 0%	-0.3 ± 0.1%

Evaluation of dosimetric impact by respiratory motion

**Figure 5:**
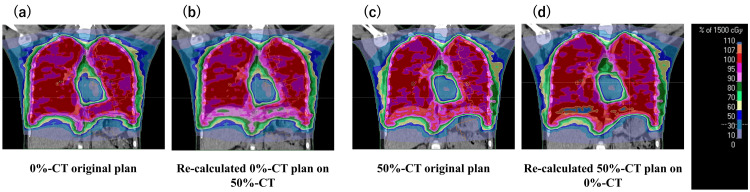
Comparison of dose distribution for 2-IC VMAT plans by different respiratory phases used for the treatment planning CT series (a) and (c) show the dose distributions of the original plan with 0% and 50% phase CT. (b) shows the plan generated with 0% phase CT that was recalculated with 50% phase CT, and (d) is vice versa.

Figure 5 shows dose distributions of the 2-IC VMAT plan due to the difference in the respiratory phase used on the treatment planning CT series. Figure 5 (a, b) shows the plan generated with 0% phase CT that was recalculated with 50% phase CT, and (c, d) is vice versa. Table 3 shows the mean and SD of dose-volume data due to the difference in the respiratory phase used in the treatment planning CT series. Due to differences in diaphragm position, the dose in the 0% phase plan was lower and the 50% phase CT plan was higher than the original plan. When the 0% phase CT plan was recalculated with the 50% phase CT, the high-dose indices (D_2%_, D_max_) showed little change compared with the original plan. Conversely, when the 50% phase CT plan was recalculated with a 0% phase CT, D_2%_ and D_max_ increased by approximately 5% and 8%, respectively.

**Table 3:** Comparison of dose-volume data for PTV caused by the difference in the respiratory phase used for the treatment planning CT series (mean and SD) VMAT, volumetric-modulated arc therapy; IC, isocenter; HI, homogeneity index; SD, standard deviation; D_X%_, the dose to percentage volumes; D_max_, the maximum dose; PTV, planning target volume

Plan (CT Phase)	Dosimetric parameters	Original plan	Recalculated plan	Relative difference (%)
1-IC VMAT (0% - CT)	D_50%_ (cGy)	1500.9 ± 1.6	1482.9 ± 10.7	-1.20 ± 0.65
	D_98%_ (cGy)	1386.3 ± 12.8	1328.6 ± 28.6	-4.17 ± 1.34
	D_2%_ (cGy)	1562.2 ± 3.9	1557.3 ± 7.2	-0.31 ± 0.30
	HI	0.1172 ± 0.0098	0.1542 ± 0.021	31.25 ± 8.98
	D_max_ (cGy)	1633.9 ± 12.2	1657.9 ± 38.5	1.46 ± 1.90
1-IC VMAT (50% - CT)	D_50%_ (cGy)	1501.1 ± 0.7	1518.9 ± 9.5	1.18 ± 0.62
	D_98%_ (cGy)	1387.6 ± 26.9	1401.0 ± 27.6	0.97 ± 0.28
	D_2%_ (cGy)	1562.1 ± 5.5	1635.7 ± 26.6	4.71 ± 1.55
	HI	0.1163 ± 0.0205	0.1545 ± 0.0315	32.72 ± 10.61
	D_max_ (cGy)	1640.7 ± 7.7	1780.9 ± 41.1	8.53 ± 2.04
2-IC VMAT (0% - CT)	D_50%_ (cGy)	1501.9 ± 1.3	1485.6 ± 10.1	-1.08 ± 0.66
	D_98%_ (cGy)	1409.5 ± 15.2	1346.7 ± 33.9	-4.47 ± 1.55
	D_2%_ (cGy)	1550.1 ± 4.9	1545.9 ± 8.5	-0.27 ± 0.29
	HI	0.0936 ± 0.0123	0.134 ± 0.0255	42.53 ± 12.02
	D_max_ (cGy)	1616.9 ± 19.5	1633.4 ± 29.9	1.03 ± 1.65
2-IC VMAT (50% - CT)	D_50%_ (cGy)	1500.6 ± 2.0	1743.1 ± 47.5	1.08 ± 0.57
	D_98%_ (cGy)	1404.6 ± 15.1	1743.1 ± 47.5	1.40 ± 1.23
	D_2%_ (cGy)	1555.1 ± 10.1	1743.1 ± 47.5	5.02 ± 1.60
	HI	0.1003 ± 0.0153	1743.1 ± 47.5	38.11 ± 15.57
	D_max_ (cGy)	1617.6 ± 31.1	1743.1 ± 47.5	7.77 ± 2.56

## Discussion

WLI is widely used as a treatment option for lung metastasis of Wilms tumor, Ewing sarcoma, and rhabdomyosarcoma and has shown improved survival rates. The European Intergroup Ewing Sarcoma Study trial reported that five-year overall survival was better in patients with than in those without WLI (61% vs. 49%, p = 0.036) [2]. A United Kingdom Children’s Cancer Study Group reported that relapse-free survival was 79.2% in patients with Wilms tumor who received WLI compared with 53.3% in patients who did not receive it [3]. Complications of WLI include decreased lung function, pneumonia, thoracic failure to thrive, and effects on cardiac function. Pein et al. reported that the risk of heart disease from radiation to the heart was 4.40 times higher in patients who received >20 Gy to the heart than in those who did not receive radiation therapy and 6.48 times higher in patients who received >5-20 Gy [8]. Guldner et al. reported that cardiac disorder and cardiac failure risks were increased linear (26% per Gy) and linear-quadratic (19% per Gy) functions of the mean heart radiation dose [16].

Kalapurakal et al. demonstrated the feasibility and dosimetric advantages of WLI-IMRT given concurrently with chemotherapy in pediatric tumors with lung metastases [10]. To the best of our knowledge, this is the first report that compared dose distributions of WLI between the AP-PA, static-field IMRT, and VMAT. In the present study, static-field IMRT had a superior cardio-sparing effect over AP-PA fields, as demonstrated by Kalapurakal et al. [10]. In addition, we confirmed the dosimetric advantages of VMAT over the AP-PA and static-field IMRT, including superior cardiac-sparing and dose homogeneity with fewer hot spots over 107% of the prescribed dose. This finding was similar to those in previous reports that compared full-arc VMAT and AP-PA fields for a single patient [11,12]. In a dosimetric comparison between 1-IC and 2-IC VMAT plans, 2-IC VMAT had a favorable point of view of heart protection (Dmean and V4.5Gy), target coverage and homogeneity, and low-dose spreading than 1-IC VMAT.

Although the 2-IC partial VMAT was proposed in this study due to mechanical limitations, obtaining an optimal dose distribution may be a better planning method. The leaves in the Varian MLC travel on a carriage to extend their movement across the field. However, the maximum leaf out of the carriage distance is only 14.5 cm [17]. Therefore, when treating a large target with a large field, optimization calculation is limited by the mechanical limitation of the MLC. For WLI in adolescents and adults, designating one lung as the target for each beam is recommended to maximize the performance of the linear accelerator. Although a general comparison cannot be made, the following factors differ from those of our case that most probably resulted in a higher dose reduction in cardiac dose parameters than that of previous studies: (a) beam arrangement and techniques, (b) PTV margin, (c) total dose, and (d) patient age [10-12,14]. Large-cohort studies including children, adolescents, and adults using standardized procedures should be conducted to validate our results.

In multi-IC plans, patient positioning errors can cause substantial deviations from the planned dose if the separation between the IC changes [18]. As shown in Figure 4, profiles were taken along the center of the PTV from the RL direction through the junction area where the two arcs meet and overlap each other. This plan demonstrates that for the right and left lung arcs, the dose distribution becomes a more constant sloping profile. Therefore, the dose distribution robustness in the joint region due to misalignment and body movement would be ensured, as shown in Table 2.

To our knowledge, this is the first report demonstrating that the WLI dose distribution changes significantly near the diaphragm because of differences in the respiratory phase. When the inspiratory CT plan was recalculated with expiratory CT, the D_98%_ value decreased by approximately 4% (Table 2). In contrast, when the expiratory CT plan was recalculated with inspiratory CT, the D_max_ and D_2%_ values increased by approximately 8% and 5%, respectively. This would be attributed to the fluence changes due to density differences near the diaphragm. In the case of IMRT planning on the inspiratory CT, to deliver a dose to a low-density region, the optimization algorithm creates a high photon fluence. Resultantly, if the diaphragm is present in that region on exhalation, a dose higher than planned will be delivered. The underdose of the inspiration would be due to the opposite phenomenon. However, the dosimetric impact of this hot or cold spot is actually smaller compared with the planned because it is averaged over the respiratory cycle. In a clinical case, 2-IC VMAT planned on inspiratory CT was employed to prevent localized overdose; however, this decision should be made based on the policies of one’s institution.

The 2-IC VMAT plan would complicate the treatment and increase the effort. However, the estimated beam-on time would not significantly change whether the IC has one or two. The mean and SD of estimated total beam-on times for 1-IC and 2-IC VMAT plans directly retrieved from the beam properties in the radiation treatment planning system were 213 ± 4 s and 212 ± 4 s, respectively. Compared to static-field IMRT, which was conventionally used for cardiac-sparing WLI, the treatment time would generally be shorter. Therefore, it was estimated that the actual treatment was five minutes longer than with a single IC plan, with only additional setup and imaging time due to the two ICs. Since this was a case of a 14-year-old patient, the patient’s patience and cooperation made the extended treatment time, not a major problem and completed the treatment course as planned. In the treatment of pediatric cancer, an increased treatment time may cause patient distress and body movements by spending long periods on the radiotherapy couch. As a result, motion suppression and sedation may become necessary, and there is a risk of shifting the treatment position. Therefore, such risks should be significantly reduced, and techniques with short treatment times are required. VMAT is a desirable technique for cardiac-sparing WLI but not necessarily the best plan, and the plan quality (technique and the number of beams and ICs) should be considered based on the patient’s condition.

This report had some limitations that should be considered. First, the follow-up was relatively short, and the treatment and side effects in the long follow-up period after the WLI with VMAT were not validated. Additionally, it is limited to only one case report for treatment. Second, because of the rarity of the case, the irradiation technique was validated in six patients who were not pediatric cancer patients. Third, the risk of secondary malignancies of normal tissues due to low-dose spillage by IMRT remains a potential problem. Fourth, the introduction of IMRT to organs with respiratory motion may cause unintended dose distribution due to the interplay effect. These findings will require clarification in future studies.

## Conclusions

The partial arc VMAT technique for cardiac-sparing WLI resulted in a significant heart dose reduction compared with conventional AP-PA and static-field IMRT. Therefore, VMAT should be considered as an effective treatment option. In addition, 2-IC VMAT is an effective option to avoid mechanical constraints during irradiation for large volumes. Consideration must be given to the CT phase used for treatment planning as dose distribution changes depending on the displacement of the diaphragm position due to respiration.
